# A case report of telitacicept in the treatment of immunocompromised and refractory lupus nephritis

**DOI:** 10.3389/fimmu.2025.1676486

**Published:** 2025-11-20

**Authors:** Yongda Lin, Chunling Liao, Zhensheng Yang, Tianbiao Zhou

**Affiliations:** Department of Nephrology, the Second Affiliated Hospital of Shantou University Medical College, Shantou, China

**Keywords:** lupus nephritis, telitacicept, systemic lupus erythematosus, biopharmaceutical, biological agent

## Abstract

Lupus nephritis (LN) is a manifestation of kidney damage in systemic lupus erythematosus and is more common in Asian populations. The standard-of-care (SOC) for LN includes antimalarials, glucocorticoids, and immunosuppressants. The efficacy and safety of biologics have been validated, and they play an important role in the treatment of LN. This case report describes a patient with immunocompromised refractory LN who had repeated severe infections after SOC and visited the clinic for recurrence of LN combined with pneumonia. The patient was admitted to the hospital this time due to “recurrent rash for 15 years, edema for 2 years, and aggravation for 1 month”. After anti-infective therapy, the treatment plan for glucocorticoids combined with telitacicept was individualized based on the patient’s immune status, and the disease was quickly controlled. Simultaneously, it demonstrated efficacy and safety during follow-up, suggesting that telitacicept may be a new treatment option for refractory LN.

## Introduction

Systemic lupus erythematosus (SLE) is a systemic autoimmune disease characterized by inflammation and immune-mediated injury to multiple organ systems, including the kidney and mucocutaneous, musculoskeletal, and hematologic systems (1). The prevalence of SLE in China is 30-70/100, 000, and it is estimated that there are 1 million patients with SLE in China, ranking first in the world in total and second in incidence (2). Approximately 50% of Chinese patients with SLE have renal impairment, and the prevalence of lupus nephritis (LN) is higher in Asian patients with SLE than in Caucasian patients (3, 4). Despite improved treatment options, 5-20% of patients with LN develop end-stage renal disease within 10 years of the initial diagnosis of SLE. Multiple comorbidities associated with immunosuppressive therapy, including infection, osteoporosis, and cardiovascular and reproductive effects, remain concerning (5). The standard-of-care (SOC) for LN includes antimalarials, glucocorticoids, and immunosuppressants.

SLE pathogenesis is complex and involves both innate and adaptive immune cells. The distinctive features of SLE are the production of autoantibodies, the formation of immune complexes that lead to organ damage, and the autoantibodies produced by B cells that play a key role (6). The recurrence rate of lupus nephritis is 40% (7, 8). For patients who have relapsed, the KIDGO guidelines recommend that the same initial therapy or recommended replacement therapy be used when LN relapses after achieving a complete or partial response. For patients with recurrent LN, a triple immunosuppressive regimen of belimumab plus glucocorticoids and mycophenolic acid analogs (MPAA) or reduced-dose cyclophosphamide may be preferred (9). Belimumab inhibits B cell proliferation by blocking the binding of soluble B-lymphocyte stimulator (BLyS) to B cell receptors and is the world’s first approved biologic for the treatment of SLE. Telitacicept binds to and neutralizes the activity of two cell signaling molecules, BLyS and a proliferation-inducing ligand (APRIL), thereby inhibiting the development and survival of plasma cells and mature B cells, and is also approved for the treatment of SLE (10). This article reports a case of telitacicept in the treatment of immunocompromised and refractory LN.

This thesis follows the CARE guidelines to write the case report and submitted file named CARE-Checklist-English-2013.

## Case report

### Patient information

A 29-year-old woman was diagnosed with “LN” 15 years ago due to facial erythema, rash, and edema of both lower limbs. She had been treated with glucocorticoids and immunosuppressants, including cyclophosphamide (CTX), during which she was hospitalized in a local hospital for multiple recurrences or pulmonary infections, including five hospitalizations for severe pneumonia. The patient was admitted to the hospital this time due to “recurrent rash for 15 years, edema for 2 years, and aggravation for 1 month”. One month ago, the patient experienced swelling in both lower extremities again, which gradually worsened. It was accompanied by chest tightness after activity, coughing, and a small amount of white phlegm. After admission, the highest blood pressure was monitored at 181/127 mmHg. The blood pressure was controlled after the addition of levamlodipine and sacubitril valsartan sodium tablets. The patient underwent bilateral hip replacement due to bilateral femoral head necrosis five years ago. Menarche occurred at 13 years of age, with a menstrual cycle of 5 days (duration) and an interval of 28–30 days. Menstruation is regular, with moderate flow, normal color, and no dysmenorrhea. The results of the ancillary examinations are presented in **Table 1**.

**Table 1:** The main examination results of the patient.

Project	Result	Reference interval	Project	Result	Reference interval
Hb g/L	85	115-150	IgA g/L	1.01	0.82-4.53
PLT 10^9/L	97	125-350	IgG g/L	5.55	7.51-15.6
Cre μmol/L	84.6	44-133	IgM g/L	1.32	0.46-3.04
UREA mmol/L	6.92	2.6-7.5	C3 g/L	0.4	0.79-1.52
ALT U/L	13	7-40	ACA IgM	(+)	(-)
AST U/L	15	13-35	anti-ssDNA	(+)	(-)
HS-CRP mg/L	40.12	0-4	anti-dsDNA	(+)	(-)
PCT ng/ml	0.24	0-0.05	ANA	(+)	(-)
U-PRO	3+	(-)	anti-SM	(-)	(-)
UOB	1+	(-)	anti-Ro/SSA	(-)	(-)
U-PRO/U-Cre mg/g	10367	0-200	anti-La/SSB	(-)	(-)

Hb, hemoglobin; PLT, platelet; Cre, Creatinine; ALT, Alanine aminotransferase; AST, Aspartate aminotransferase; HS-CRP, Hypersensitive C-reactive protein; PCT, Procalcitonin; U-PRO, Urinary protein; UOB, Urine Occult Blood; U-PRO/U-Cre, Urinary protein/Urinary; IgA, Immunoglobulin A; IgG, Immunoglobulin G; IgM, Immunoglobulin M; C3, Complement C3; ACA IgM, Anticardiolipin antibody IgM; anti-ssDNA, anti-single-stranded DNA; anti-dsDNA, anti-double-stranded DNA; ANA, antinuclear antibody; anti-SM, Anti-Smith Antibody

Key physical examination indicates Weight 54kg, T 36.7°C, P 101 bpm, R 20 bpm, Bp 181/127mmHg, full moon face, pale skin and mucous membranes all over the body, moderate anemic appearance, no rash all over the body, symmetrical normal respiratory movement, regular respiratory rhythm, clear sounds on percussion of both lungs, coarse breathing sounds of both lungs No obvious dry or wet rales were heard. No obvious abnormalities were found in the physical examinations of the heart, lungs and abdomen. Moderate edema in both lower extremities.

### Renal biopsy pathology report

**Figure 1:**
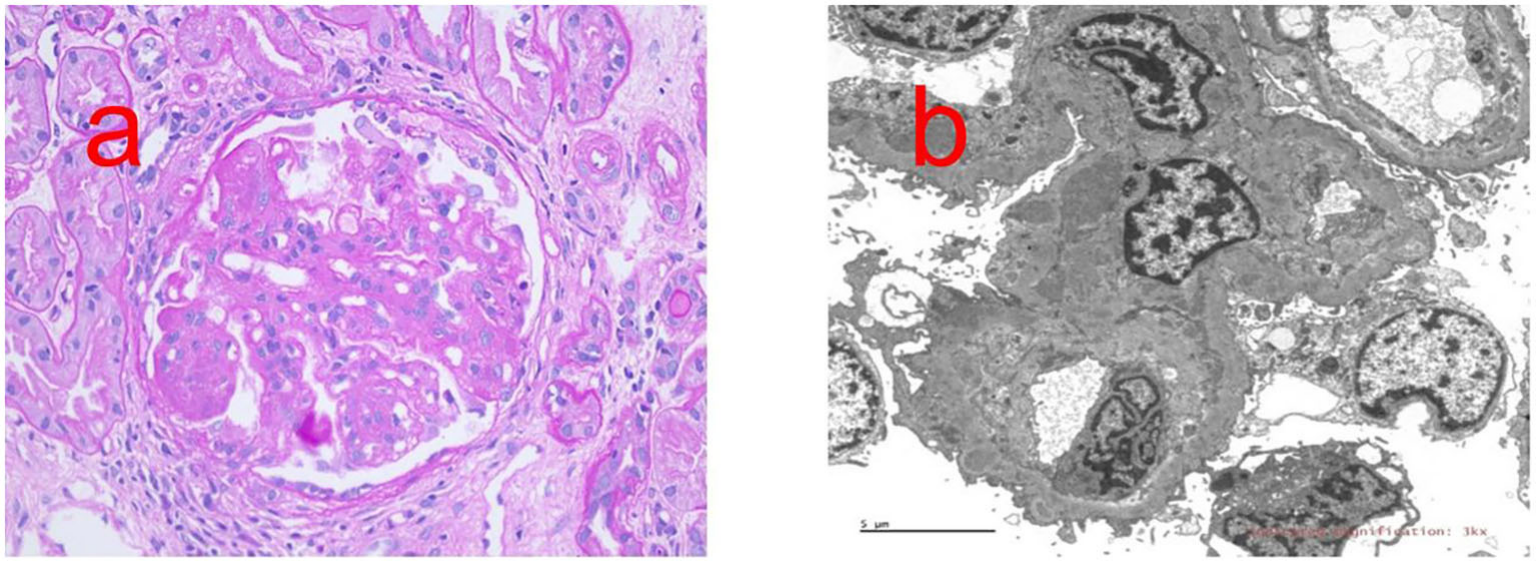
Renal biopsy report. **(a)** PAS staining shows Diffuse spherical hyperplasia of glomerular mesangial cells and endothelial cells (×400). **(b)** Transmission electron microscope shows the podocytes of the visceral epithelial cells are fused, and electron-dense deposits can be seen under the endothelium and mesangial cells and stroma proliferate.

Proliferative LN with membranous LN (class IV + V); activity index(AI), 10/24; chronic index (CI), 2/12; immunofluorescence, IgG (2+), IgM (1+), IgA (1+), C3 (3+), and c1q (2+) (**Figure 1**).

### SLEDAI score

Fifteen points (Heme-granular or RBC urinary casts, Hematuria, Proteinuria, Low complement, High DNA binding, and PLT <100 x 10^9/L).

### Treatment plan and therapeutic effect

**Figure 2:**
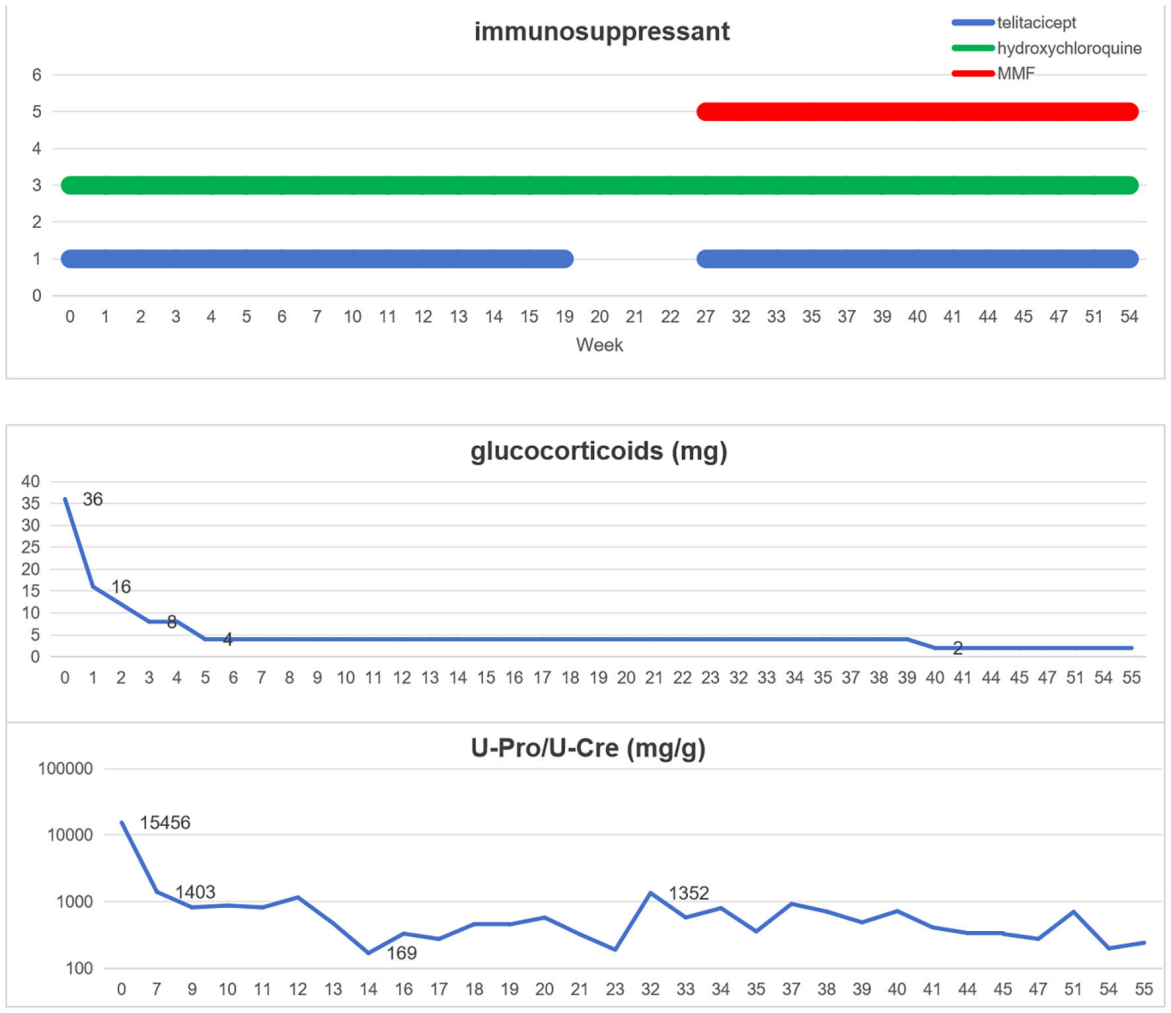
The treatment plan and efficacy.

This case was a patient with recurrent LN with severe glucocorticoid complications, multiple previous severe pulmonary infections. The report results of the patient’s lymphocyte subsets showed that the patient had significantly low levels of T lymphocytes, B lymphocytes, and NK cells, suggesting that the patient had low innate and adaptive immunity (**Table 2**). At the same time, this patient also had elevated hypersensitive C-reactive protein, because he had pulmonary infection. After controlling the pneumonia infection through anti-infection treatment, considering the patient’s low immunity, we formulated a personalized treatment plan. The treatment plan and its efficacy are shown in **Figure 2**.

**Table 2:** Flow cytometry lymphocyte subset test report.

Lymphocyte subsets	Count(Number/μL)	Reference interval(Number /μL)
CD3^+^ (T lymphocyte)	318	955 - 2860
CD3^+^CD4^+^CD8^-^ (CD4+T lymphocyte)	134	550 - 1440
CD3^+^CD4^-^CD8^+^ (CD8+T lymphocyte)	177	320 - 1250
CD3^+^CD4^+^CD8^+^ (CD4+CD8 + T lymphocyte)	1	6 - 56
CD3^+^CD4^+^CD8^-^/CD3^+^CD4^-^CD8^+^	0.76	0.7 - 2.8
CD56^+^CD16^+^CD3^-^ (NK cells)	15	150 - 1100
CD56^-^CD19^+^CD3^-^ (B lymphocyte)	32	90 - 560

Telitacicept, 80 mg/160 mg ih (intramuscular injection) qw (once a week), was administered, and the dosage of methylprednisolone was reduced from 36 mg per day at the time of hospitalization to 16 mg and rapidly reduced to 4 mg within 5 weeks. After six weeks of telitacicept treatment, the urine protein/creatinine ratio significantly decreased from 15, 455 mg/g to 1, 403 mg/g, and after 12 weeks of treatment, it decreased to 169 mg/g. After 20 weeks of treatment, telitacicept was discontinued due to the patient’s economic factors. During this period, the monitored urine protein level gradually increased to 1, 352 mg/g. In the 27th week, methylprednisolone (4 mg) was continued and MMF was added in combination with telitacicept, 80/160 mg ih qw, for treatment. In week 40, methylprednisolone was adjusted to 4 mg orally every other day, and MMF was combined with telitacicept (80 mg ih q2w(twice a week)/qm (once a month).

This patient with systemic lupus complicated with lupus nephritis had a history of up to 15 years and was hospitalized for repeated recurrences during the treatment period. During this period, the patient underwent SOC and experienced adverse reactions after treatment, including multiple severe pneumonia infections, femoral head necrosis and obesity. Due to repeated treatment with immunosuppressants, the patient has a weakened immune system.The SLEDAI score of, 15 points, for this recurrence and the renal biopsy report indicated active LN (class IV + V), immunological high activity (anti-dsDNA +, C1q 2+, C3 3+), and concurrent pneumonia infection. Therefore, it is necessary to select a safe and effective treatment regimen with low dosages of glucocorticoids. The application of immunosuppressants can reduce the dosage of hormones and increase the disease remission rate. Therefore, we choose to add telitacicept to the treatment plan. Telitacicept plus SOC therapy seems to be an appropriate regimen. However, considering the patient’s low immunity, we developed an individualized treatment plan for low-dose glucocorticoids in combination with telitacicept and hydroxychloroquine. After 6 weeks of treatment, partial response was achieved, and after 12 weeks of treatment, complete response was achieved. After treatment remission, the follow-up for 1 year was completed, the patient’s urine protein control was stable, no infectious complications occurred during the whole treatment period, only a small dose of glucocorticoids (5 mg Once every two days) was maintained, and glucocorticoid side effects such as moon face disappeared.

## Discussion

The traditional treatment of SLE is dominated by pan-targeted anti-inflammatory and immunosuppressive drugs, including antimalarials, glucocorticoids, and immunosuppressants, which alone or in combination are the SoCs for SLE. Currently, the treatment regimen is mainly selected according to the pathological type of LN in clinical practice. For patients with class I/type II LN, no specific immunosuppressive therapy other than immunosuppressive therapy is required for extrarenal lupus. Active type III./IV (with or without type V) LN is a progressive disease that requires prompt and effective immunosuppressive therapy to control immunoinflammatory lesions in renal tissue, block the progression of renal injury, and prevent the formation of renal fibrosis. The main treatment methods include I. glucocorticoids combined with MPAA, II glucocorticoids combined with low-dose intravenous CTX, III glucocorticoids combined with belimumab and MPAA or low-dose intravenous CTX (triple immunization), and IV. MPAA in combination with calcineurin inhibitor (CNI) (multi-target protocol). Combination regimens of immunosuppressants targeting different targets have become a new therapeutic strategy to control LN activity to improve treatment response rates, reduce relapse rates, and reduce drug-related adverse events by lowering the dose of corticosteroids and other immunosuppressants (9, 11).

For patients who have relapsed, the KIDGO guidelines recommend that the same initial therapy or recommended alternative therapy (9) be used when LN relapses after achieving a complete or partial response. For patients with recurrent renal disease, a triple immunosuppressive regimen of belimumab plus glucocorticoids, MPAA, or reduced-dose CTX may be preferred. Belimumab blockade of Blys can attenuate the autoimmune response without interfering with the host’s innate defenses (12). The results of the BLISS-LN subgroup analysis of East Asian populations also showed that the degree of anti-dsDNA and anti-C1q decline and complement elevation was more significant in patients with LN who were anti-ds-DNA, anti-C1q-positive, or hypocomplementemic in the SOC combined with belimumab group than in the SOC combined with placebo group (13). Rituximab can directly deplete B cells and takes effect more quickly than belimumab and telitacicept. The 2024 KDIGO guidelines recommend that for the treatment of active LN, rituximab can be considered for patients with continuous disease activity or those who do not respond adequately to initial conventional treatment (9). The LESIMAB study (14) included 116 patients with refractory systemic lupus erythematosus, seventy-three (62.9%) patients achieved a response at six months. However, the article reports that the Serious infection rate during the treatment process was 12.6/100 patient-years. Telitacicept, a dual-target biologic that targets BLyS and APRILs, has also been approved for the treatment of SLE (10). At week 48 of tetaercept in patients with active systemic lupus erythematosus, 75.8% of patients achieved SRI-4 response in the 240 mg tetatercept group, 68.3% in the 160 mg group, 71.0% in the 80 mg group, and 33.9% in the placebo group, and the rates of adverse effects and events were comparable to those in the placebo group (15).

The application of biological agents has increased the remission rate of refractory lupus nephritis while reducing the use of hormones. In this case, the patient’s lupus nephritis recurred again. Considering the patient’s history of femoral head necrosis, biological agents need to be part of the treatment plan. However, considering that this patient had repeated severe infections in the past, and the lymphocyte subsets suggested low innate and adaptive immunity. Therefore, the risk of infection became the primary consideration indicator for choosing biological agents. Due to the excessive infection risk associated with rituximab, we compared the infection risk during the treatment with telitacicept and belimumab. Multiple studies simultaneously demonstrated the safety of telitacicept and belimumab, especially in terms of the risk of infection (16, 17). An observational study showed that over 24 weeks of treatment, patients receiving telitacicept had a significantly higher probability of achieving lupus low disease activity state compared to those receiving belimumab (18). Another study also showed that the LLDAS attainment rate was higher in the telitacicept group (54.86% vs. 33.13%) (16).

Multiple studies have confirmed the efficacy and safety of telitacicept in SLE patients. Chen et al. (19) reported the case of a patient with refractory proliferative LN treated with telitacicept. The patient was treated with a combination of glucocorticoids (5 mg/day), hydroxychloroquine, MMF, and telitacicept (160 mg once a week) at 19 months of follow-up, and the massive proteinuria was controlled. Li et al. (20) reported that patients with LN developed pneumonia after treatment with glucocorticoids, MMF, and CNI. Telitacicept combined with low-dose MMF was administered, and proteinuria was relieved quickly without secondary infection. During the patient’s hospitalization this time, Hypersensitive C-reactive protein was found to be elevated, and imaging suggested pneumonia. Therefore, we did not add MMF in the treatment. Shockingly, the patient still achieved complete remission rapidly. After the treatment remission, a one-year follow-up was completed, and the patient’s urine protein was stably controlled. No infectious complications occurred throughout the treatment period, and only a small dose of glucocorticoids was maintained. In this case, a personalized treatment plan was provided based on the patient’s personal disease activity, immune status, and previous complications, achieving good therapeutic effects. Unfortunately, the patient had suspended the use of telitacicept for several weeks due to economic problems, which led to a slight increase in urine protein levels.

## Conclusion

The treatment plan for glucocorticoids combined with telitacicept was individualized based on the patient’s immune status, and the disease was quickly controlled. Simultaneously, it demonstrated efficacy and safety during follow-up, suggesting that telitacicept may be a new treatment option for refractory LN.

## Data Availability

The datasets presented in this study can be found in online repositories. The names of the repository/repositories and accession number(s) can be found in the article/supplementary material.
